# Prediction of disease progression indicators in prostate cancer patients receiving HDR-brachytherapy using Raman spectroscopy and semi-supervised learning: a pilot study

**DOI:** 10.1038/s41598-022-19446-4

**Published:** 2022-09-06

**Authors:** Kirsty Milligan, Xinchen Deng, Ramie Ali-Adeeb, Phillip Shreeves, Samantha Punch, Nathalie Costie, Juanita M. Crook, Alexandre G. Brolo, Julian J. Lum, Jeffrey L. Andrews, Andrew Jirasek

**Affiliations:** 1 Department of Physics, University of British Columbia, Kelowna, BC Canada; 2 Department of Statistics, University of British Columbia, Kelowna, Canada; 3 Trev and Joyce Deeley Research Centre, BC Cancer, Victoria, BC Canada; 4 Department of Radiation Oncology, University of British Columbia, Kelowna, BC Canada; 5 Department of Chemistry, University of Victoria, British Columbia, Canada; 6 Department of Biochemistry and Microbiology, University of Victoria, Victoria, Canada

**Keywords:** Cancer metabolism, Tumour biomarkers, Tumour virus infections, Biological physics, Cancer metabolism, Urological cancer, Cheminformatics, Metabolomics, Cheminformatics, Bioanalytical chemistry, Medical and clinical diagnostics

## Abstract

This work combines Raman spectroscopy (RS) with supervised learning methods—group and basis restricted non-negative matrix factorisation (GBR-NMF) and linear discriminant analysis (LDA)—to aid in the prediction of clinical indicators of disease progression in a cohort of 9 patients receiving high dose rate brachytherapy (HDR-BT) as the primary treatment for intermediate risk (D’Amico) prostate adenocarcinoma. The combination of Raman spectroscopy and GBR-NMF-sparseLDA modelling allowed for the prediction of the following clinical information; Gleason score, cancer of the prostate risk assessment (CAPRA) score of pre-treatment biopsies and a Ki67 score of < 3.5% or > 3.5% in post treatment biopsies. The three clinical indicators of disease progression investigated in this study were predicted using a single set of Raman spectral data acquired from each individual biopsy, obtained pre HDR-BT treatment. This work highlights the potential of RS, combined with supervised learning, as a tool for the prediction of multiple types of clinically relevant information to be acquired simultaneously using pre-treatment biopsies, therefore opening up the potential for avoiding the need for multiple immunohistochemistry (IHC) staining procedures (H&E, Ki67) and blood sample analysis (PSA) to aid in CAPRA scoring.

## Introduction

Prostate cancer is the second most commonly diagnosed cancer in men and accounts for 10% of all cancer deaths in Canada^1^. When prostate cancer is suspected, the most common method of diagnosis is tissue biopsy^2^. Tissue biopsies are then assessed histologically to determine both Gleason score^3^ and although not currently routine procedure, the number of Ki67 positive cells in the tissue is often measured, both of which are markers of disease aggression^4^. Gleason score has long since been the gold standard used to judge metastatic potential and ultimate risk of prostate cancer death^5–7^. The original Gleason grading system was developed in the 1960s, however, more recently (2005) the International Society of Urologic Pathology (ISUP) devised a modified method of the original Gleason grading system. This modified Gleason grading system was created with the aim to improve consistency of diagnosis amongst pathologists and more accurately assess disease prognosis^8,9^. More recently, Gleason grade grouping has also been used as a prognostic indicator, wherein grade group 1 is the least aggressive and usually slow growing and grade group 5 is the most aggressive and can progress very quickly. This method has been particularly beneficial in distinguishing between Gleason score 7 (3 + 4) (grade group 2) and Gleason score 7 (4 + 3) (grade group 3) disease. For example, disease which presents as mostly pattern 4 with some pattern 3 abnormalities may not progress exactly as disease which is mainly pattern 3 with some pattern 4 abnormalities, although both cases would be described as Gleason score 7^6^.

Ki67 is a protein associated with cellular proliferation in all phases of the cell cycle with the exception of quiescent cells. Increased cellular proliferation is a hallmark of cancer cells and increased expression of Ki67 may correlate with aggressiveness of disease. Berlin et al.^10^ demonstrated, using meta-analysis of 21 separate studies comprising of a total of 5419 patients, that high (>6%) Ki67 levels in prostate cancer was strongly associated with poorer clinical outcome when compared with patients with lower levels of Ki67 expression. Despite this, Ki67 is not routinely used as a marker of proliferation in the prognostic evaluation of prostate adenocarcinoma. There is evidence that consideration of Gleason score and Ki67 score simultaneously could provide better prognostic information. For example, Fisher et al.^11^ demonstrated that multivariate analysis of Gleason score, Ki67 scores and prostate specific antigen (PSA) levels provided significantly more prognostic information in a sample cohort of 293 patients than univariate analysis of each marker individually. The subjectivity of both Gleason and Ki67 scoring^12^, as well as a need for a combined approach which considers multiple clinical factors simultaneously makes the development of a reliable and robust method of assessing, simultaneously, the aforementioned clinical indicators of disease desirable in order to obtain a more accurate prognosis and plan treatment accordingly.

Prostate specific antigen (PSA) is a glyco-protein routinely used as a marker for screening and early detection of prostate cancer. PSA is expressed in both normal and neoplastic prostate tissue. As well as being used as a marker for early detection of prostate cancer, PSA is also used to monitor the likelihood of disease recurrence^13^ and is thought to be critical in monitoring treatment response^14^. Normal levels of serum PSA vary with age. The population median for men at age 40 is 0.6 ng/ml and by age 60 is 1.1 ng/ml^15–17^, however there are many non disease related factors which alter PSA levels such as body mass index (BMI)^18,19^, recent sexual activity, bicycle riding^20,21^, race^22,23^, medications^24,25^ and genetic polymorphisms^19,26^. Despite the variation in PSA levels due to non-cancer related factors, PSA remains the cornerstone in monitoring disease progression in patients with prostate cancer. There are various measures of PSA kinetics post RT which are used to monitor disease recurrence and metastasis, predominantly, PSA velocity^27,28^, doubling time^29,30^ and slope^31^. However, more recently there has been significant debate concerning which measure of PSA following RT is the most reliable in terms of predicting recurrence^13^. It has been demonstrated that at 4 years after treatment a PSA < 0.2 ng/ml can be used to define cure for men who have been treated with low dose rate brachytherapy. PSA levels should be used in conjunction with other prognostic factors such as Ki67 scores^11,32^ as using PSA levels alone as an indication of disease aggressiveness can be misleading^33–35^.

Cancer of the prostate risk assessment (CAPRA) score was developed by The University of California San Francisco (UCSF) in 2005 to provide a more accurate assessment of risk criteria in prostate cancer. A CAPRA score is valid across multiple treatment approaches e.g. prostatectomy, RT, hormonal therapy and watchful waiting and it predicts an individual’s likelihood of metastasis, cancer-specific mortality, and overall mortality. The factors used to calculate CAPRA score are; age and PSA (ng/ml) at diagnosis, Gleason score, T stage and percentage of positive biopsy cores. It is calculated in a similar manner as the D’Amico classification, yet generally considered to out-perform D’Amico classification in terms of risk stratification^36–40^.

Raman spectroscopy (RS) is a label-free, non-invasive optical technique which results in a unique "fingerprint" spectrum, congruent with the composition of bio-chemicals present in the sample under interrogation. RS is can be used to acquire information on multiple biochemicals simulataneously^41^ and is desirable from a clinical standpoint as it is non-destructive to samples^42^. RS has been used in combination with chemo-metrics to study both cellular response to external stimuli e.g. ionising radiation and the effect this has on cell metabolism^43–45^. The field of RS for bio-analysis and radiation response monitoring has advanced toward *in vivo* analyses of cells, in order to better understand the tumour environment^46–48^. However, there has been little advancement in RS applications in a clinical setting, in particular, the area of RS as a tool to better understand and predict patient response to radiation therapy.

This study demonstrates the power of RS combined with group and basis restricted non-negative matrix factorisation (GBR-NMF)^49,50^ and linear discriminant analysis (LDA) as a tool for the prediction of Gleason and CAPRA score in the pre-treatment biopsies of patients receiving high dose rate brachytherapy as a sole treatment for intermediate risk (D’Amico) prostate adenocarcinoma. Furthermore, Ki67 scores in post-treatment biopsies could also be predicted using the RS coupled with GBR-NMF-sparseLDA method using the spectra acquired from pre-treatment biopsies. The major advantage of this method for the prediction of disease progression in clinical samples is the ability to predict multiple clinical factors simultaneously using the same Raman spectra acquired from a single sample, with reasonable accuracy. Furthermore, the ability to predict post-treatment clinical information using pre-treatment samples, could be enormously beneficial in a clinical setting. For example, obtaining information on how Ki67 scores will look post-treatment, before HDR-BT has commenced, opens up the ability to plan alternative treatments or more efficient combination therapies. Combining RS and GBR-NMF modelling with machine learning strives toward the ability to predict response to RT on an individual basis and reduces inter-user variability with regards to histological evaluation of tissue, which has been shown to have a significant effect on the integrity of prognosis in prostate cancer^12^. The RS method also improves analysis time and resources, especially if Gleason and Ki67 scores could be predicted using RS with reasonable accuracy, reducing the need for IHC staining which is subjective and time consuming.

## Results

### Prediction of Gleason score

**Figure 1:**
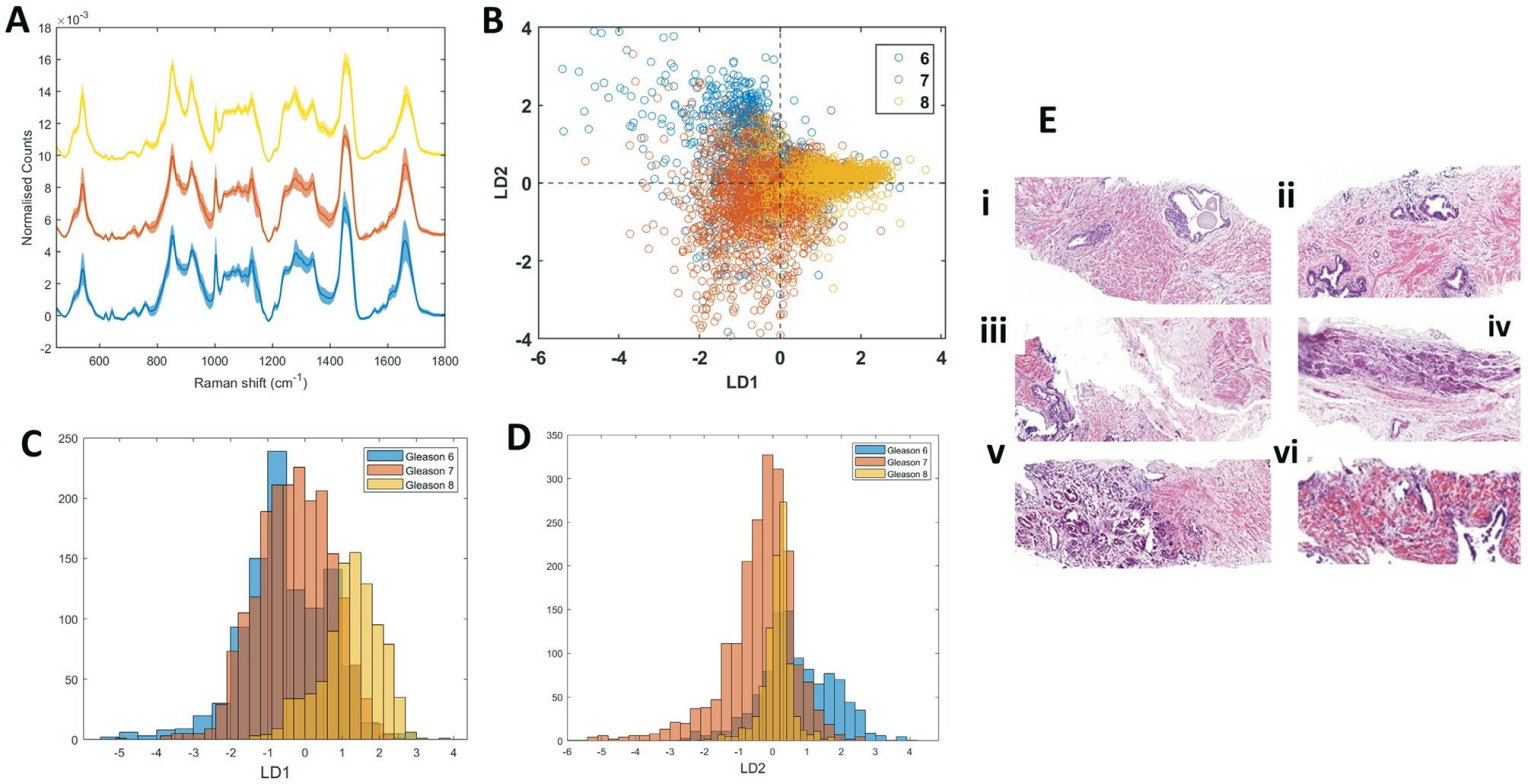
(**A**) Average Raman spectrum of Gleason score 6 tissue (blue), Gleason score 7 tissue (orange) and Gleason score 8 tissue (yellow). Shadow spectrum represents + /− 1 standard deviation. Spectra were acquired using a Renishaw InVia Raman microscope (100 × objective, 785 nm excitation, 30 s acquisition time and 0.45 mW power). Spectra are baseline corrected, normalised and smoothed using a Savitsky-Golay filtering algorithm. (**B**) scatter plot of linear discriminant 1 (LD1) and linear discriminant 2 (LD2) scores obtained from RS-GBR-NMF-sparseLDA classification of RS acquired from Gleason score 6 (blue points), 7 (orange points) and 8 (yellow points). (**C**) Histogram of LD1 scores obtained from RS-GBR-NMF-sparseLDA classification of RS acquired from Gleason score 6 (blue points), 7 (orange points) and 8 (yellow points). (**D**) Histogram of LD2 scores obtained from RS-GBR-NMF-sparseLDA classification of RS acquired from Gleason score 6 (blue points), 7 (orange points) and 8 (yellow points). (**E**) Pre-treatment (LEFT) and post-treatment (RIGHT) (single dose) haematoxylin and eosin (H&E) stained images of patients 76 (i,ii), 86 (iii,iv) and 88a (v,vi).

Eligible patients for HDR-BT as a sole treatment had intermediate risk disease (D’Amico classification) based on initial diagnostic work up. Pathology reports on the pre-treatment biopsies provided information on % of biopsy area showing adenocarcinoma, percentage of Gleason pattern 4 within the tissue as well as overall Gleason score. The 9 patients had Gleason scores ranging from 6 to 8. Initial diagnostic biopsies assigned all individuals in the study as Gleason score 6 or 7, however, subsequent protocol-directed biopsies revealed that individual 86 in fact displayed a Gleason score of 8 (4+4). As the protocol-directed biopsies matched the cores used to obtain RS data, individual 86 was assigned into the Gleason score 8 group in this study, therefore this individual can be considered high risk. The average Raman spectrum acquired from individuals with Gleason score 6, 7 and 8 disease is shown in Figure 1a as a blue, orange and yellow spectrum, respectively. Shadow regions represent +/− 1 standard deviation. Visually, the spectra obtained from Gleason score 6, 7 and 8 tissue appears markedly similar. The biochemical scores obtained from GBR-NMF modelling of the pre-treatment RS for each individual were used in sparseLDA classification of the spectra as Gleason score 6, 7 & 8. The number of variables (biochemical scores) used in the model was reduced to 8 in order to avoid over-fitting. Application of GBR-NMF-sparseLDA modelling, revealed that stratification of Gleason score can be achieved using scores obtained on; co-enzyme A, cysteine, glyceryl tripalmitoleate, methionine, histidine, isoleucine, glutathione and oleic acid. The scatter plot of the linear discriminants (LDs) obtained from the sparseLDA model is shown in Fig. 1B, wherein it can be seen that LD1 provides separation between Gleason score 8 spectra from Gleason score 6 and 7 and LD2 provides separation between Gleason score 6 and 7. It is expected that Gleason 8 which is pure pattern 4 disease will be distinct from Gleason score 6 which is pure pattern 3. Gleason 7, on the other hand is a combination of pattern 3 and 4 with variable amounts of pattern 4. The more pattern 4 present, the more it should resemble Gleason 8, both metabolically and clinically. This is further demonstrated by the histogram plot of LD1 and LD2 scores shown in Fig. 1C,D, respectively. An example of the haematoxylin and eosin (H&E) images which were used to perform Gleason grading of the tissue are shown in Fig. 1E (i,iii & v) for patients 76, 86 and 88a, respectively.

The model was validated by removing a randomly selected 25% of the data from the training set and using this as a test set. The resultant classification is shown in Table 1. The overall accuracy of the model was 66%.

**Table 1:** RS-GBR-NMF-sparseLDA classification of Gleason score (test data).

	Prediction		
Observed	GS6	GS7	GS8
GS6	**123**	47	4
GS7	130	**402**	81
GS8	25	56	**133**

### Prediction of CAPRA score

CAPRA score was calculated using points assigned to: age at diagnosis, PSA at diagnosis, Gleason score of the biopsy, clinical stage and percent of biopsy cores involved with cancer (based on CAPRA score calculator provided by UCSF Department of Urology, https://www.urology.ucsf.edu).

**Figure 2:**
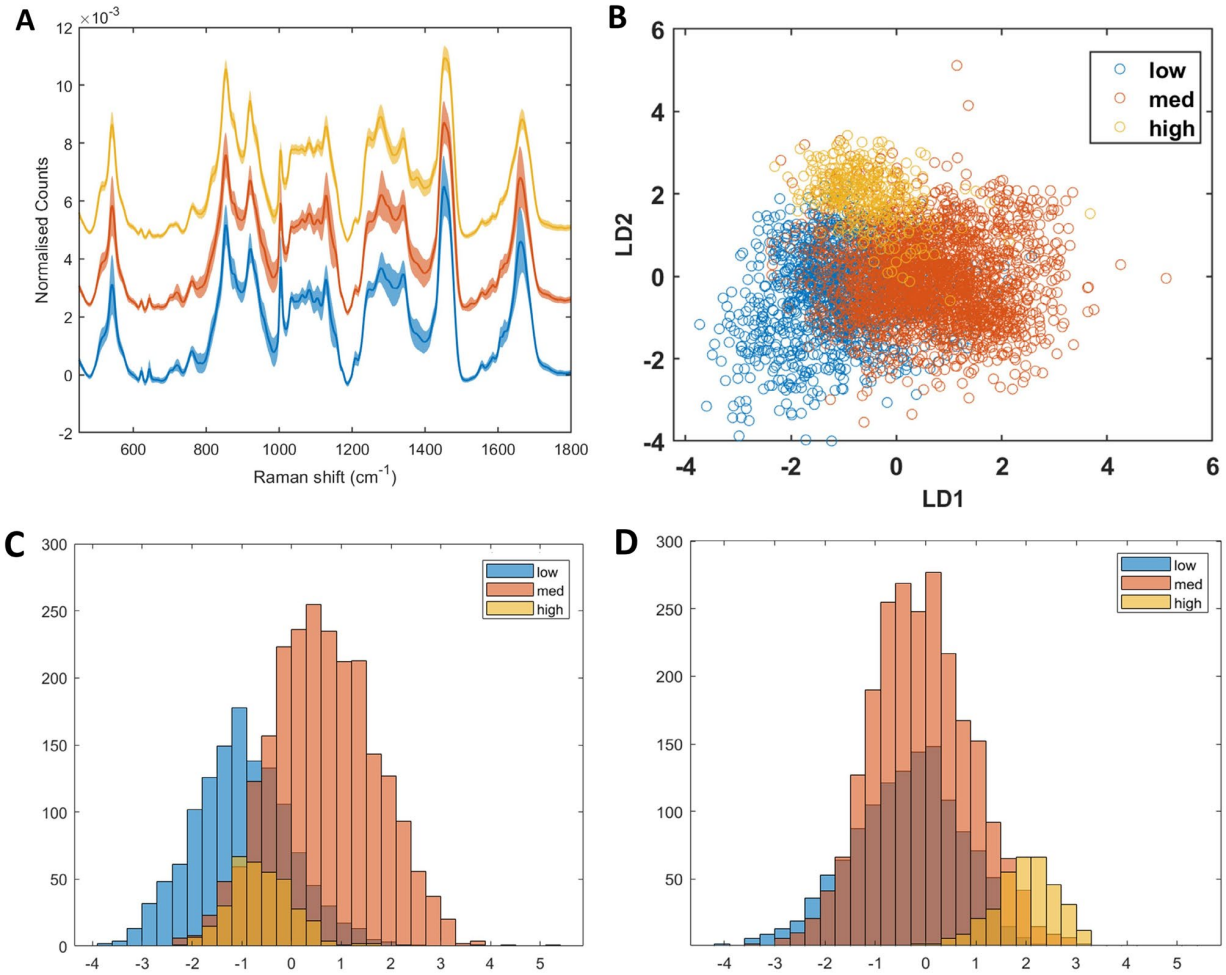
(**A**) Average Raman spectrum of CAPRA low group (blue), CAPRA medium (orange) and CAPRA high (yellow). Shadow spectrum represents + /− 1 standard deviation. (**B**) scatter plot of linear discriminant 1 (LD1) and linear discriminant 2 (LD2) scores obtained from RS-GBR-NMF-sparseLDA classification of RS acquired from Gleason score 6 (blue points), 7 (orange points) and 8 (yellow points). (**C**) Histogram of LD1 scores obtained from RS-GBR-NMF-sparseLDA classification of RS acquired from CAPRA low (blue points), CAPRA medium (orange points) and CAPRA high (yellow points). (**D**) Histogram of LD2 scores obtained from RS-GBR-NMF-sparseLDA classification of RS acquired from CAPRA low (blue points), CAPRA medium (orange points) and CAPRA high (yellow points).

The RS-GBR-NMF-sparseLDA model was applied to the classification of the spectra into "low" (CAPRA score of 0-2), "medium" (CAPRA score of 3-5) and "high" (CAPRA score of 6-10). The average Raman spectrum acquired from individuals with low, medium and high CAPRA scores is shown in Fig. 2A as a blue, orange and yellow spectrum, respectively. Shadow regions represent +/− 1 standard deviation. As with Gleason score, the average RS acquired from the three CAPRA groups appeared very similar. However, application of GBR-NMF-sparseLDA modelling, again revealed that separation of CAPRA score can be achieved using scores obtained on; co-enzyme A, cysteine, glyceryl tripalmitoleate, methionine, histidine, isoleucine, glutathione and oleic acid. The scatter plot of the linear discriminants (LDs) obtained from the sparseLDA model is shown in Fig. 2B, wherein it can be seen that LD1 provides separation between CAPRA low and medium groups and LD2 provides some separation between CAPRA low and medium from the CAPRA high group. This is further demonstrated by the histogram plot of LD1 and LD2 scores shown in Fig. 2C,D, respectively.

The classification capability of the RS-GBR-NMF-sparseLDA model to classify CAPRA low, medium and high scores was assessed based on a training-testing setup. The model was validated by removing a randomly selected 25% of the data from the training set and using this as a test set. The resultant classification is shown in Table 2. The overall accuracy of the model was 70%.

**Table 2:** RS-GBR-NMF-sparseLDA classification of CAPRA score (test data).

	Prediction		
Observed	Low	Med	High
Low	**32**	17	8
Med	17	**157**	73
High	41	131	**487**

### Prediction of Ki67 in post treatment samples

**Figure 3:**
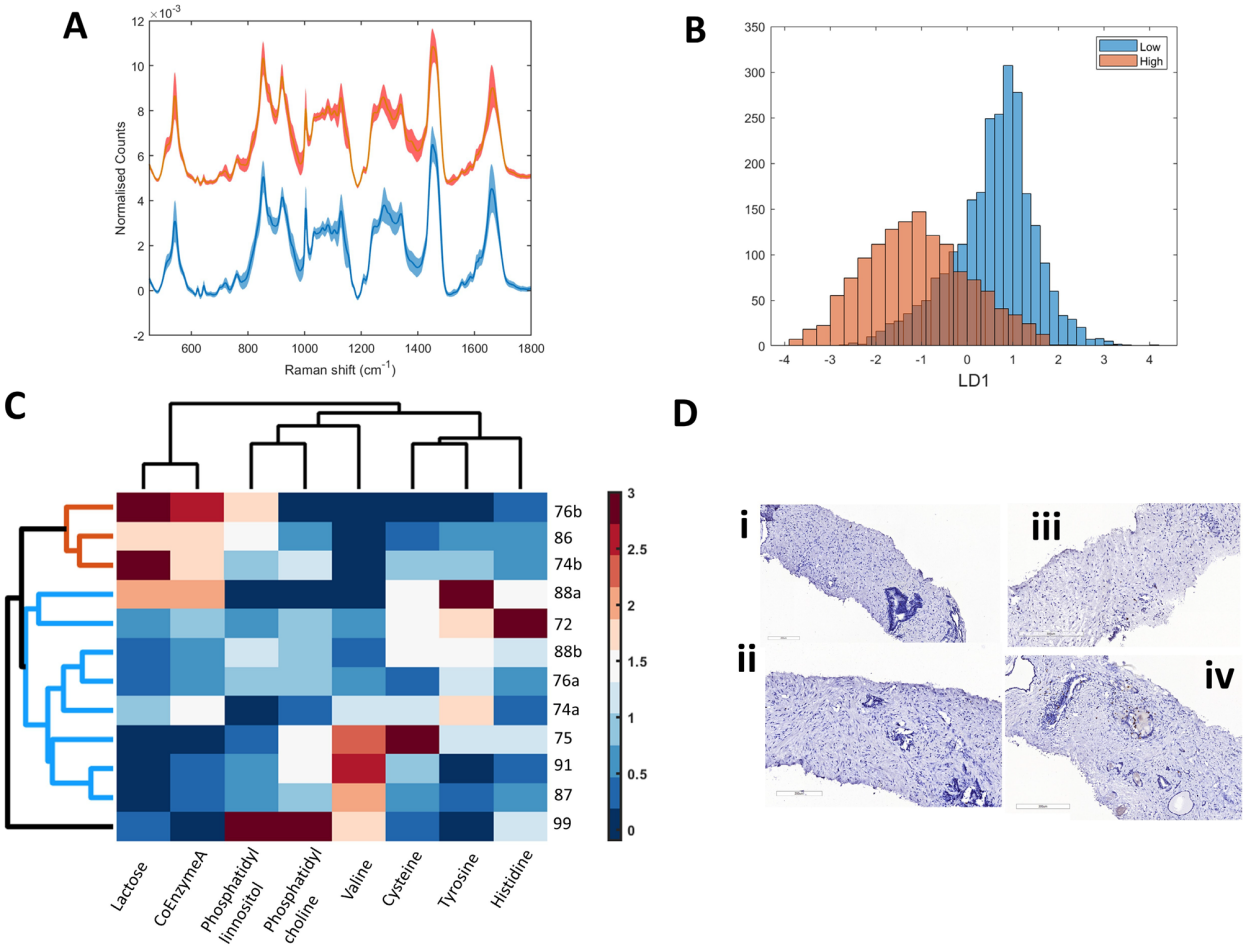
(**A**) Average Raman spectrum of Ki67 low tissue (blue), Ki67 high tissue (orange). Shadow spectrum represents + /− 1 standard deviation. (**B**) Histogram of LD1 scores obtained from RS-GBR-NMF-sparseLDA classification of RS acquired from Ki67 low spectra (blue points) and Ki67 high spectra (orange points). (**C**) HCA analysis of the average score for each individual biopsy on the biochemicals listed on the x-axis. Branch height represents closeness of a particular sample to another. Shorter branches represent samples which are more similar than samples linked via taller branches. The biopsies largely cluster in to Ki67 low (blue branches) and Ki67 high (orange branches) groupings, with the exception of individual 99. The coloured pixels represent the average score each of the 8 biochemicals for the individual biopsy, represented as binned into 5 levels. The dark blue pixels represent little or no expression of a particular biochemical, whereas the dark red pixels represent high levels of a particular biochemical within the sampled tissue. (**D**) Pre-treatment (LEFT) and post-treatment (RIGHT) (single dose) Ki67 stained images of patients 76 (i,ii) and 86 (iii,iv).

Ki67 scores were measured as a percentage of tumour cells which stained positive for Ki67 in post-treatment biopsies and values for each individual are stated in Table 5. To assess prediction capabilities of Ki67 scores in post-treatment samples using Raman data acquired from pre-treatment biopsies, the RS-GBR-NMF-sparseLDA model was applied to the pre-treatment data. Post-treatment Ki67 scores were split into two groups, <3.5% (low group) and >3.5% (high group). The average pre-treatment spectra from the resultant groups is shown in Fig. 3A (low = blue spectrum, high = orange spectrum). Application of sparseLDA classification of the GBR-NMF scores in to these two groups, yielded significant separation along LD1, as shown in the histogram of LD1 scores in Fig. 3B (low group = blue, high group = orange). The biochemicals selected for optimal separation between the two classes were; lactose, co-enzyme A, phosphatidylinnositol, phosphatidylcholine, valine, cysteine, tyrosine and histidine. The mean score obtained from GBR-NMF modelling for each of the 8 selected biochemicals is shown as a pixel chart in Fig. 3C. Dark red pixels represent a greater score for any one biochemical whereas dark blue pixels represent a lower score. This plot represents the expression profile of the selected 8 biochemicals. Hierarchical cluster analysis (HCA) was performed using the euclidean distance and linkage method of the mean score on the 8 biochemicals across the 12 pre-treatment biopsies. Dendrograms on the y-axis represent identified clusters. The lesser the height of the dendrogram, the more closely the biopsies are related in terms of the expression profile of the 8 biochemicals used in the sparseLDA modelling. For example, samples 91 and 87 were most similar, and sample 99 is the least similar to the other biopsies in terms of biochemical expression.

The samples largely clustered together in to two main groups, Ki67 "low" group (blue cluster) and Ki67 "high" group (orange cluster). Patient 99 did not fall in to either cluster and exhibited a different biochemical expression profile, mostly due to high levels of phospholipids. Ki67 scores were obtained from IHC staining of post-treatment biopsies as shown in Fig. 3D. Figure 3D (i & ii) shows an example of two Ki67 "low" post-treatment biopsies (patient 72 and patient 91, respectively) and Fig. 3D (iii & iv) shows two Ki67 "high" biopsies (patient 76b and patient 99, respectively).

The model was validated using a training and testing setup, as described for both Gleason score and CAPRA score, the data was split into training (75%) and testing (25%). The resultant classification accuracy for the test set is shown in Table 3. The overall accuracy of the model was 85%.

**Table 3:** RS-GBR-NMF-sparseLDA classification of Ki67 score post-treatment (test data).

	Prediction	
Observed	Low	High
Low	**287**	61
High	84	**514**

To assess the robustness of the model, a modified training-testing method was used, wherein the data was split in to a training and testing set wherein the test set comprised all of the RS acquired from an individual biopsy. This method was chosen to assess whether identifiable patient differences in spectra were responsible for the classification. Table 4 lists the % classification accuracy for each test set for each individual biopsy.

**Table 4:** Accuracy of classification of the spectra acquired each individual, measured as a percentage of spectra classified correctly as Ki67 low or Ki67 high.

Biopsy	% Accuracy of classification
72	93
74a	80
74b	57
75	98
76a	88
76b	91
86	52
87	93
88a	60
88b	86
91	85
99	15

## Discussion

The Raman spectra acquired from pre-treatment biopsies from 9 individuals were analysed using a GBR-NMF model^49^, wherein scores were obtained pertaining to the relative contribution of 31 individual bio-chemicals known to be present within human tissue and cells (listed in Table A1). The scores obtained for each of the biochemicals was subsequently used in a sparse linear discriminant analysis (sparseLDA) for classification of Gleason score, CAPRA score and Ki67 score (<3.5% or >3.5% in post-treatment samples). The RS-GBR-NMF-sparseLDA model allowed for both prediction of the clinical parameters listed above and identification of the bio-chemicals which were important in the respective classifications.

This study has demonstrated the use of RS as a tool to obtain multi-parametric information from tissue biopsies acquired pre-HDR-BT treatment for prostate adenocarcinoma. Using RS combined with GBR-NMF-sparseLDA modelling, we have shown that it is possible to stratify individuals based on their Gleason score, CAPRA score and post-treatment Ki67 score. This model allowed us to stratify the patients in the study according to three separate clinical indicators of disease progression, using data acquired in a single acquisition. This is advantageous from a clinical standpoint as this method has the potential to replace the need for multiple IHC staining procedures. Arguably, another advantage of the RS-GBR-NMF-sparseLDA model is the reduction in user subjectivity that is often observed when IHC is the method employed to provide prognostic information. For example, Gleason score may vary between pathologists^12,51^, which is why central review of pathology by a specialised uro-oncology pathologist is recommended^52,53^. Our RS-GBR-NMF-sparseLDA model did not provide perfect stratification between Gleason score 6 and Gleason score 7 patients. However, the model allowed us to identify co-enzyme A, cysteine, glyceryl tripalmitoleate, methionine, histidine, isoleucine, glutathione and oleic acid as the most important biochemicals in making some distinction between Gleason score 6 and Gleason score 7 tissue. Gleason score 8 tissue displayed good separation from Gleason score 6 and Gleason score 7 based on these biochemicals. Previous studies by Penney et al*.*^54^ also identified glutathione as an important metabolite in distinguishing between Gleason score 6 disease and Gleason score 7 or higher, using mass spectrometry of serum samples. They concluded that glutathione was more abundant in low grade disease as opposed to high grade disease, likely due to glutathione involvement as a primary cellular antioxidant which is known to play a key role in carcinogenesis and is also known to be involved in cellular response to oxidative stress caused by chemotherapeutic drugs^26,55–58^. Other metabolites identified by the RS-GBR-NMF-sparseLDA model to be important in distinguishing between Gleason scores 6-8 were methionine and histidine. A study by Stabler et al.^59^ found an association between serum methionine and cysteine levels and aggressiveness of prostate cancer in a clinical investigation involving 58 subjects. It is therefore likely that the RS-GBR-NMF-sparseLDA model supports the assumption that methionine and cysteine levels are in fact related to aggressiveness of disease, as both of these amino acids were selected as important features in stratifying Gleason score 6, 7 and 8 disease. We speculate that the significance of co-enzyme A, glyceryl tripalmitoleate and oleic acid in stratifying the samples based on Gleason score, could be due to elevated expression of fatty acid synthase (FAS), which has been previously postulated to be a marker of tumour aggressiveness^60^.

Although there have been studies which have shown that RS can be used to stratify samples based on Gleason grading^61–63^, to the best of our knowledge, this is the first demonstration of how RS can be used to predict both Gleason and CAPRA score in pre-treatment samples, as well as predict post-treatment indicators of disease progression, e.g. Ki67 score.

Cancer of the prostate risk assessment (CAPRA) score is a measure of disease risk classification and is used to aid in clinical decision making. There are many methods used to evaluate prostate cancer risk, including the D’Amico classification for which all our patients were initially classified as intermediate risk. However, CAPRA score was developed by The University of California San Francisco (UCSF) to improve upon the accuracy of the D’Amico classification. CAPRA score is calculated in a similar manner to D’Amico classification but generally accepted to provide better accuracy in predicting likelihood of disease recurrence^38^. CAPRA score was calculated for each individual biopsy, using the following clinical indicators; age, PSA at diagnosis, Gleason Score, clinical stage and percentage of positive biopsy cores. The resultant three groupings; low, medium and high, were predicted using the RS-GBR-NMF-sparseLDA model and the prediction accuracy using the "leave-one-out" approach is shown in Table 4 for each individual. Figure 2B shows that although the average RS acquired from the low, medium and high CAPRA score groupings (Fig. 2A, blue, orange and yellow spectrum, respectively) are visually very similar, application of the RS-GBR-NMF-sparseLDA model using scores (obtained on the following biochemicals; cysteine, DNA, glyceryl tripalmitoleate, histidine, isoleucine, lactose, methionine and oleic acid) provides separation of the spectra according to those groupings. It is perhaps unsurprising that 6 of the 8 chemicals selected by the RS-GBR-NMF-sparseLDA model to stratify individuals based on CAPRA score also stratify the patients based on Gleason score, as CAPRA score is calculated partially based on Gleason score. It is therefore likely that a lot of the stratification observed in Fig. 2B is a result of separation along Gleason score. However, the RS-GBR-NMF-sparseLDA model appears to provide better separation of CAPRA score as opposed to Gleason score. There is greater separation of the "low", "medium" and "high" CAPRA groups, whereas Gleason score 6 and Gleason score 7 samples proved difficult to achieve maximal separation using this model. This is particularly interesting as it suggests that there may be more biochemical information contained within the tissue samples that pertains to CAPRA score, than Gleason score alone, e.g. the difference in biochemical composition of the tissue is better represented by the CAPRA scoring system than by Gleason grading alone. DNA and lactose were also selected as important variables in distinguishing between CAPRA score, however the reason for this is unclear and further work would have to be undertaken in order to both validate and investigate the reason for the difference in DNA and lactose content. Results may be clouded by varying percentage of pattern 4 in the Gleason 7 biopsies. Whilst the Raman spectra for the 3 Gleason groups and CAPRA groups were visually very similar, some differences were apparent between the groups. Notably, the 1200–1460 cm^-1^ region displayed distinct spectral differences mainly between Gleason 8 compared with Gleason 6 7 groups as well as between CAPRA high compared with CAPRA low and medium groupings. The main difference in the spectra here are is the increased intensity of the peak at 1379 cm^-1^ in both the Gleason 8 spectrum and the CAPRA high spectrum compared with the less aggressive disease spectra. We speculate that this is likely due to DNA or lipid content. It has been reported in the literature that more aggressive prostate adenocarcinoma displays increased nucleic acid content^61,64–66^. However, our RS-GBR-NMF-sparseLDA model did not identify DNA in the top 8 most important biochemicals in stratifying the data based on Gleason and CAPRA score. This could potentially be due to consideration of the entire spectrum in the RS-GBR-NMF-sparseLDA model and not single peaks. Conversely, the model identified glutathione as an important biochemical in stratifying according to Gleason and CAPRA score and one of the main differences in the RS of Gleason and CAPRA groups is the peak at 1659 cm^-1^, which can likely be attributed to glutathione^67^. As previously mentioned, a study be Penney et al*.*^54^ concluded that glutathione was more abundant in low grade disease as opposed to Gleason grade 8 disease. This is in line with our observation of the RS according to Gleason and CAPRA grouping wherein the 1659 cm^-1^ peak is more intense in the lower grade disease spectra. Further investigation is warranted to verify these biochemical differences using another method.

Finally, and arguably most importantly, we have also demonstrated in this study that post-HDR-BT treatment information can be obtained from pre-treatment samples using RS combined with the GBR-NMF-sparseLDA approach. Ki67 is a nuclear protein and tumour proliferation marker. In recent years, Ki67 has attracted significant attention as an independent prognosticator of treatment outcome in patients receiving external beam radiation therapy (EBRT) for prostate cancer. For example, Cowen et al.^68^ reported that a Ki67 score of >3.5% (Ki67 high) in pre-treatment prostate cancer biopsies was strongly associated with higher rates of biochemical failure after conventional dose RT for prostate cancer. The patients in this study that presented with Ki67 scores <3.5% pre-treatment (Ki67 low), had bNED (biochemical no evidence of disease) rates of 76% as opposed to only 33% for those in the Ki67 "high" group at 5 years (*p* <0.0001). This study also concluded that Ki67 is likely linked to an intrinsic tumour aggressiveness and radiation resistance. They also found that Ki67 was linked with abnormalities in p53 metabolism and MDM2. Despite this, Ki67 is not routinely used in clinical settings as a prognostic factor in the assessment of prostate cancer, likely due to lack of standardised assays and research into appropriate cut points for risk classification^68^. A more recent study by Wilkins et al.^69^ also reported Ki67 to be an independent prognostic factor of biochemical recurrence after radiation therapy, independent of established prognostic factors such as Gleason score, PSA and stage of disease. Similarly, Pollack et al.^70^ reported Ki67 to be a strong predictor of both distant metastasis and cause specific death in 537 patients receiving RT and androgen deprivation therapy (ADT), however this study used a cut-off of 11.3% to score Ki67 as "low" or "high", given the higher risk group of the patients involved in the study. Ki67 correlation with biochemical failure and disease recurrence after HDR-BT treatment for prostate cancer is an area which is understudied and could prove valuable in providing a more accurate risk stratification and identifying potential therapeutic targets.

Based on the aforementioned studies, we investigated whether a Ki67 threshold of <3.5% (low) or >3.5% (high) in post-HDR-BT biopsies (median Ki67 score = 2.9), could be predicted using information obtained from the RS acquired on the corresponding pre-treatment biopsy. The RS-GBR-NMF-sparseLDA model was applied to chemical scores obtained from the RS of the pre-treatment samples, and the samples were classified as "low" (a Ki67 score of <3.5% in the patient’s post-treatment biopsy) or "high" (a Ki67 score of >3.5% in the patient’s post-treatment biopsy). The average Ki67 "low" and "high" Raman spectrum is shown in Fig. 3A. Visually, there were very few identifiable differences between the Ki67 low and Ki67 high RS. As with the classification of both Gleason score and CAPRA score, LD1 provided clear separation between the Ki67 low (blue) and Ki67 high (orange) spectra (Fig. 3B). Validation of the model was performed by separating the data into a training (75%) and testing set (25%) as well as removing all spectra acquired from 1 individual from the training set. This method was chosen to assess whether identifiable patient differences in spectra were responsible for the classification. The model performed with reasonable accuracy on the blind test sets, with the lowest % accuracy of classification 52% 4 (with the exception of patient 99). The biochemicals which are responsible for the separation of Ki67 low and Ki67 high spectra were included in HCA, shown in Fig. 3C. HCA clusters data points together based on the euclidean distance of each variable used in the analysis, ultimately forming a hierarchy of clusters showing which samples are most similar and which samples are least similar in terms of biochemical expression. Performing HCA on the biochemical scores of lactose, co-enzyme A, phosphatidylinnositol, phosphatidylcholine, valine, cysteine, tyrosine and histidine provides a metabolic profile of these biochemicals across each pre-treatment biopsy. For example, it can be deduced from the branch height on the y-axis, that biopsies belonging to individuals 87 & 91 are most similar in terms of their expression of the biochemicals listed along the x-axis. Conversely, the biopsy obtained from individual 99 is least similar in terms of expression of the aforementioned 8 biochemicals to any of the other samples. The HCA also shows that the biopsies largely cluster in to Ki67 low (blue branches) and Ki67 high (orange branches) groupings, with the exception of individual 99. The coloured pixels represent the average score each of the 8 biochemicals for the individual biopsy, represented as binned into 5 levels. The dark blue pixels represent little or no expression of a particular biochemical, whereas the dark red pixels represent high levels of a particular biochemical within the sampled tissue. Figure 3C shows that the clustering of the biopsies into Ki67 low and Ki67 high can largely be attributed to high levels of co-enzyme A and lactose in the Ki67 high group, whereas in the Ki67 low group the expression of these biochemicals is lower. Likewise, in the Ki67 high group there is generally higher levels of phospholipids (phosphatidylinnositol and phosphatidylcholine), and amino acids (valine, cysteine, tyrosine & histidine), whereas in the Ki67 low group, the levels of those biochemicals tends to be lower. This finding is congruent with the findings reported by Keshari et al.^71^, using NMR spectroscopy, that Ki67 expression is higher in more aggressive disease and also correlates with increased levels of phospholipids in the tumour tissue, specifically phosho-cholines. The reason for this is not clear, but it has been speculated that increased phospholipids in more advanced disease could be due to stromal-epithelial interactions driving prostate cancer progression^72,73^ and in response to therapy^74^. Increased phospholipid expression in prostate cancer is not observed in cell culture and this further supports the assumption that stromal-epithelial interactions and the tumour microenvironment play a key role in both the progression of prostate cancer and the response to treatment. The predictive accuracy for each individual as Ki67 low or Ki67 high is listed in Table 4. As the classification accuracy of the spectra acquired from each individual is relatively high (>52%, for all individuals with the exception of individual 99), the RS-GBR-NMF-sparseLDA method could potentially eliminate the need for post-treatment IHC if Ki67 is a useful prognosticator for treatment success in HDR-BT treatment for prostate cancer. This would also allow for post-treatment information to be available and acquired from pre-treatment tissue samples. This could potentially provide foresight into how an individual may respond to treatment before that treatment takes place, opening up potential pathways to alternative treatment options or more effective combined treatments. It is likely that individual 99 displayed poor classification accuracy (15%) due to high levels of phospholipid expression (not observed with the remaining Ki67 high individuals, and low levels of co-enzyme A and lactose (as shown in HCA clustergram in Fig. 3C). The reason for this is unclear and would require further investigation.

This study had several limitations, in that the sample size is relatively small (n=9), and the classifications were based around IHC staining and interpretation by a single pathologist. Whereby, subjectivity can lead to inaccurate results. Another drawback to this study is the lack of spatial correlation between the RS and tissue substructures from H&E stained images. As part of ongoing work, a larger sample size should be investigated, wherein RS can be grouped according to tumour/stroma and assessed accordingly. The main aim of this study was to highlight that RS combined with a GBR-NMF-sparseLDA model, can predict and corroborate histopathology analyses without prior knowledge of area of region of acquisition, as well as provide biochemical information on what distinguishes between those classes. This could be achieved by setting a threshold of percentage of spectra from any individual belonging to a particular class. For example, as shown in Table 4, nine out of twelve biopsies displayed >60% of the total spectra obtained classified correctly as Ki67 low or high, therefore setting a threshold of 60% would have resulted in the correct prediction of 9 patients as Ki67 low or high, increasing the threshold to 80% would have resulted in eight of the twelve biopsies being correctly classified. Further work would be required to validate this approach and the likelihood of misclassification in a larger cohort of patients is essential and currently ongoing. The metabolic profiles identified here which correlate with Gleason score, CAPRA score and Ki67 score (post-treatment) should also be validated using another method such as mass spectrometry (MS) or NMR spectroscopy, although our findings here are supported by the current literature. Additionally, we are confident in the stratification of the tissue according to Gleason score, CAPRA score and Ki67 score, and acknowledge that the model has limitations in predictive capabilities of some singular clinical indicators. For example, classification of PSA measurements (less than or greater than the median value of 4.56 ng/mL) resulted in poor classification accuracy as highlighted in Fig. A1, which is not unexpected given that PSA is a serum marker and RS was acquired from tissue samples in this case.

We believe the findings reported here, and the identification of the metabolites associated with Gleason score, CAPRA score and Ki67 score in prostate cancer could prove valuable in identifying both; new therapeutic targets for combination therapies as well as providing multiple prognostic indicators (including information on post-treatment biopsies) from data acquired from a single acquisition. The classification accuracy for each of the prognostic factors investigated was reasonably high, suggesting this method could reduce the subjectivity issues associated with IHC staining as well as reduce the need for multiple staining/interpretation procedures.

## Materials and methods

### Patient cohort

The nine patients included in this study received HDR-BT as mono-therapy for intermediate risk prostate adenocarcinoma. All individuals involved in this study provided informed consent for the use of bio-specimens and reporting of results for research purposes. All identifiable information has been omitted for each individual. All methods and procedures were carried out in accordance with the procedures and guidelines provided by Health Canada and Public Health Agency of Canada Research Ethics Board (REB# H17-02904). The study has been approved by the University of British Columbia and BC Cancer Research Ethics Board. Table 5 includes information on prognostic indicators including age, Gleason score and baseline PSA. A summary of the patient cohort is shown in Table 5. Prior multi-parametric MRI had identified the dominant lesion, the location of which was transferred to transrectal ultrasound (TRUS) images through rigid registration for the purpose of targeting the lesion for both biopsy and dose escalation. HDR BT dose prescription was 27 Gy in 2 fractions, given in 2 separate procedures 2 weeks apart. Dose escalation to the dominant lesions aimed for 140-150% of prescription. Biopsies were collected prior to fraction 1 for initial baseline evaluation and again 2 weeks later, subsequent to a single dose of 13.5 Gy, immediately prior to delivery of fraction 2 (13.5 Gy). Biopsies were taken under anaesthesia, transperineally with template and ultrasound guidance based on the prior fusion, prior to the first treatment and 2 weeks later of the same area, prior to the second treatment. Samples were immediately placed in hypothermosol and hand delivered to UBCO. Paired samples were sent for haematoxylin and eosin (H&E) staining and examination at KGH pathology to confirm tumour presence and accurate targeting. Ki67 staining was performed at BC Cancer Victoria.

**Table 5:** Summary of disease markers in patient cohort receiving HDR-BT for prostatic adenocarcinoma.

	Age	Gleason score	CAPRA risk group	% pattern 4	% Ki67 + (post-treatment)	PSA (ng/mL)
72	65	7	Medium	20–30	1.5	8.5
74a	64	7	Medium	20	0	4.56
74b		7		20	5.9	
75	66	6	Low	0	3.1	3.34
76a	65	6	Low	0	2.7	1.6
76b		6	Low	0	5.8	
86	73	8	High	100	7.9	16.84
87	64	7	Medium	5–10	3.2	6.2
88a	59	6	Medium	0	1.3	9.13
88b		7		90	0.8	
91	62	6	Low	0	2.1	3.54
99	72	7	Medium	80	5.2	4.02

### Sample collection and preparation for RS

Samples were harvested by radiation oncologists as needle core biopsies preserved in hypothermosol (Thermo Fisher Scientific, MA, USA). The biopsies were embedded in mounting medium (Tissue-Plus™ O.C.T. Compound, Fisher Scientific, MA, USA), snap frozen in liquid nitrogen and stored at − 80°.

### Spectral acquisition

Needle core biopsies (approximately 0.1 × 0.5 cm) from prostatic adenocarcinoma tumours were sectioned in to 20 micron thick slices using a rotary cryostat (HM 550; MICROM International GmbH, Walldorf, Germany) and placed on magnesium fluoride slides. Prior to Raman analysis, spectra were allowed to air dry for 10 minutes.

Spectra were acquired using a Renishaw InVia Raman microscope (Gloucestershire, U.K.) with a 100× dry objective (NA 0.9) (Leica Microsystems, Wetzlar, Germany). All spectra were acquired using a 785 nm diode laser (0*.*5mW/µm^3^ laser power density at sample, 30 second acquisition time). For each biopsy slice analysed, 3 randomly selected regions were chosen (without prior knowledge of disease/non disease regions) and Raman spectra acquired from a 120 micron squared area, using 15 micron step size (8 8 grid). This was repeated for 2–3 slices per patient for each pre-treatment biopsy, resulting in a total of 380 spectra per biopsy and 3905 spectra in total. Spectra were acquired over the range of 450–1800 cm^−1^. The number of slices analysed per biopsy as well as the total number of spectra obtained after processing are listed in Table 6.

**Table 6:** Summary of RS data acquired from each individual.

	Pre/post treatment	Number of slices analysed	Total number of spectra (after processing)
72	Pre	2	335
74a	Pre	2	337
74b	Pre	2	325
75	Pre	2	346
76a	Pre	2	233
76b	Pre	2	328
86	Pre	2	346
87	Pre	2	285
88a	Pre	2	303
88b	Pre	2	342
91	Pre	2	370
99	Pre	2	355
			Total 3905

### Spectral processing and analysis

Spectra which contained cosmic rays or saturation were removed prior to spectral processing. In-house algorithms were used in the estimation and subtraction of spectral background due to fluorescence^75^ and to shift the spectra to account for calibration drifts over time (based around 1003 cm^−1^ phenylalanine peak)^76^. Background subtraction was performed using a modified version of the signal removal method described by Schulze et al.^77^ An initial estimate of the baseline was performed by applying a Savitsky-Golay filter with a window size of 7% of the total range of the data (582 points). This process provides a separation threshold, wherein data above the threshold is deemed signal and data below the threshold is deemed to be noise. Any data above the threshold is replaced with the value of the SG filter BL estimate at that point (i.e., the signal is removed). This process is then repeated on the modified data set for 20 iterations (as this number of iterations resulted in no further change of the BL estimate), providing a final estimate of the background spectrum which is then subtracted from the original data. The spectra were then normalised such that the area under the curve was equal to 1. Finally, Savitsky-Golay filtering (window size = 3, order = 1) was used to smooth the data.

Group and basis restricted non-negative matrix factorisation^49^ (GBR-NMF) was performed on the spectra in order to decompose the data matrix, X, into three lower rank matrices such that X WAS. These three matrices included the chemical bases responsible for variation in the spectra (S), a matrix responsible for scaling the bases (A), and the scores on the bases representing the contribution of each chemical to each spectrum (W). GBR-NMF modelling was carried out using publicly available code^78^ for R version ×64 3.6.1^79^, as previously reported^50^.

Linear discriminant analysis^80^ is a popular supervised classification technique which arises from several potential assump- tions on generative schemes underlying observed data^81^. One such classification approach, termed ‘optimal scoring’, lends itself particularly well for penalisation approaches, allowing the analyst to seek low-dimensional (often termed ‘sparse’) solutions to the linear discriminant analysis problem^81,82^. Analyses herein were carried out using the open source "lda" and "sparseLDA" functions in R (version ×64 3.6.1)^79^ from the “MASS”^83^ and “sparseLDA”^84^ packages, respectively. Variables were reduced from 31 to 8 using sparseLDA and a linear discriminant model was then fit using the selected 8 variables.

In order to test the predictive accuracy of the model, the data was randomly split into 75% training and 25% testing subsets. No data included in the training set was applied in the testing of the model, however as the data was randomly split, the training and testing subsets may include data from the same biopsy. The results of this method are stated in Table 1 for Gleason score, Table 2 for CAPRA score and Table 3 for Ki67. The validation steps were repeated 3 times using different training/testing combinations and did not result in error (+/− 1 standard deviation) of more than 3% in any predictive case. The Ki67 model was also tested using a leave one out cross validation approach (LOOCV) wherein all data acquired from a particular biopsy was excluded from the training set and applied as a test set only. The percentage of spectra from each biopsy correctly classified as Ki67 low or high is stated in Table 4.

## Supplementary Information

Supplementary Information.

## Data Availability

The datasets used and/or analysed during the current study are available from the corresponding author on reasonable request.

## Code availability

All codes are publicly available unless otherwise stated. In house algorithms are available upon request.
