# Supplementary material for: Prediction of disease progression indicators in prostate cancer patients receiving HDR-brachytherapy using Raman spectroscopy and semi-supervised learning: a pilot study
Source: Sci Rep. 2022 Sep 6;12:15104. doi: 10.1038/s41598-022-19446-4 (PMC9448740; doi:10.1038/s41598-022-19446-4)
Supplement: Supplementary file 1 — Supplementary Information. [file 41598_2022_19446_MOESM1_ESM.docx]

# A Supplementary Information

**Table A1.** 31-reference-biochemical Raman chemical library

Alanine Arginine Asparagine Citric acid CoEnzymeA Collagen Cysteine DNA

Glucose Glutamic acid Glutathione Glycerol

Glyceryl tripalmitoleate Glycogen

Histidine Isoleucine Lactose Mannose Methionine Oleic acid Palmitic acid Phenylalanine

Phosphatidylcholine Phosphatidylserine Phosphatidylinnositol Serine

Stearic acid Triglycerides Tryptophan Tyrosine Valine


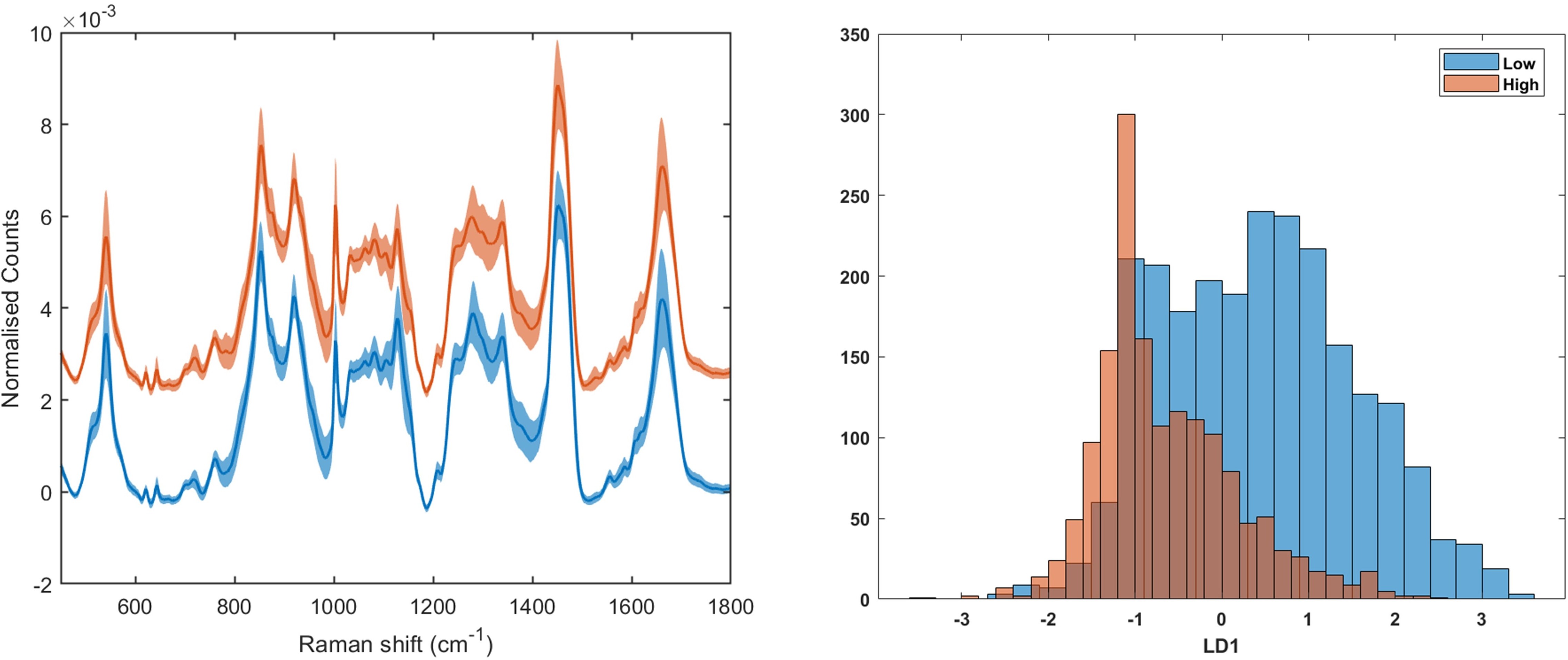


**Figure A.1. Left** Average Raman spectrum of PSA "low" (<median value of 4.56 ng/mL) tissue (blue), PSA "high" tissue (>median value of 4.56 ng/mL) (orange). Shadow spectrum represents +/- 1 standard deviation. Spectra were acquired using a Renishaw InVia Raman microscope (100x objective, 785 nm excitation, 30s acquisition time and 0.45 mW power). Spectra are baseline corrected, normalised and smoothed using a Savitsky-Golay filtering algorithm. **Right** Histogram of LD1 scores obtained from RS-GBR-NMF-sparseLDA classification of PSA acquired from PSA low spectra (blue points) and PSA high spectra (orange points). There is no significant stratification of the PSA low and PSA high groups, however, the PSA low group did exhibit a greater range of LD scores when compared with the PSA high group.
